# 

**Published:** 1879-02-20

**Authors:** 


### *BOOK NOTICES.*

PHYSIOLOGY. *Preliminary Course of Lectures* by JAMES T. WHITAKER, M.A. M.D., Professor of Physiology and Clinical Medicine at the Good Samaritan Hospital, etc.—On the influence of physiology upon practice; on the conservation of force; on the origin of life and the evolutions of its forms; and on protoplasm, bone, muscle, nerve and blood; 288 pages, illustrated.—Cincinnati, CHANCY R. MURRAY, 103 W. Sixth street, 1879.

This is an admirable work in which the popular and learned Prof. Whitaker presents "the foundation facts and principles upon which the steady edifice of physiology is built." It should be read by both student and practitioner.

DIFFERENTIAL DIAGNOSIS, *A Manual of the Comparative Semiology of the more important Diseases*, by F. DEHAVILLAND HALL, M.D., Assistant Physician to the Westminster Hospital, London. American edition with extensive additions.—Philadelphia, D. G. BRINTON, 115 Second street, 1879.

A volume of 200 pages in which diagnostic points of diseases are given in a well-defined, comprehensive plan; presented in tabular method from which comparisons may be most easily drawn, and correct conclusions deduced. It is an eminently practical and useful work.

SCRIBNER'S MONTHLY MAGAZINE is among the best and most handsomely illustrated literary journals in the world. It is filled with solid matter on religious and other topics, and has a corps of the best story writers in the country. It is the very magazine for the lady of the doctor's house. Scribner & Co., New York.

ST. NICHOLAS is the Child's Magazine, and is unrivaled in its beauty and the finish of its illustrations and stories. The boys and girls prize it as the prettiest and nicest gift that can be tendered. Subscribe for it. Scribner & Co., New York.

